# Prognostic Nomogram of Osteocarcinoma after Surgical Treatment

**DOI:** 10.1155/2022/9778555

**Published:** 2022-09-21

**Authors:** Qiuli Wu, Canchun Yang, Haolin Yan, Zheyu Wang, Zhilei Zhang, Qiwei Wang, Renyuan Huang, Xumin Hu, Bo Li

**Affiliations:** ^1^Department of Orthopedics, Tianjin Medical University General Hospital, Tianjin 300052, China; ^2^Department of Orthopedics, Sun Yat-sen Memorial Hospital of Sun Yat-sen University, Guangzhou 510120, China

## Abstract

**Purpose:**

This study aimed to establish a valid prognostic nomogram for osteocarcinoma after surgical management.

**Methods:**

Based on the SEER database, we retrieved the clinical variables of patients confirmed to have osteocarcinoma between 1975 and 2016. Then, we performed univariate and multivariate analyses and constructed a nomogram of overall survival.

**Results:**

Multivariate analysis of the primary cohort revealed that the independent factors for survival were age, grade, pathologic stage, T stage, and surgery performed. All these factors were showed by the nomogram. The correction curve of survival probability showed that the prediction results of nomogram well agreed with the actual observation results. The C index of the nomogram used to predict survival was 0.82; the AUC of 1-year, 3-year, and 5-year survival rates in the training cohort were 0.9, 0.819, and 0.80631, respectively, indicating that the model was accurate and reliable; whether the operation was performed or not; T stage; grade; and age were the main factors affecting the survival of patients. The AUC of the validation cohort for 1 year, 3 years, and 5 years were 0.8, 0.831, and 0.80023, respectively.

**Conclusion:**

The proposed nomogram can more accurately predict the prognosis of patients with osteocarcinoma after surgical management. This could be a potential method that services clinical work.

## 1. Introduction

As a common primary bone malignant tumour, osteosarcoma is from primitive osteogenic stroma. The onset age is mostly in adolescents and young people under 20 years old. Osteosarcoma is a highly malignant tumour. Its clinical feature is that it is very prone to pulmonary metastasis [1]. Studies have shown a close connection between the rapid growth of adolescent bones and the occurrence and development of osteosarcoma [2]. The aetiology of osteosarcoma primarily includes epidemiological, genetic, and environmental factors. To date, the globally recognised risk factors related to osteosarcoma progression include Paget's disease, hereditary retinoblastoma, chromosome abnormalities, and the effects of ionising radiation and alkylating agents [3]. With the development of surgical techniques and the application of neoadjuvant chemotherapy, limb-salvage surgery combined with systemic chemotherapy has become a better choice than simple amputation. These multidisciplinary combined treatments have improved the 5-year survival rate of 60%–70% amongst patients with nonmetastatic osteosarcoma. Despite the great success of osteosarcoma treatment, improvements in the survival rate of osteosarcoma patients over the past decade are very limited. Moreover, patients with tumour metastasis or recurrence have always shown poor prognosis. The 5-year overall survival (OS) of osteosarcoma patients with recurrence or metastasis is only about 20%, and it has not yet been completely solved clinically [4].

Before neoadjuvant chemotherapy, the cure rate of osteosarcoma patients treated by surgery alone is about 15%–17% [5]. In the 1970s, with the advent of neoadjuvant chemotherapy, the 5-yeardisease-free survival (DFS) rate of osteosarcoma patients has increased to 60%–70% [5]. At present, its comprehensive treatment methods include surgery, neoadjuvant chemotherapy, targeted therapy, and immunotherapy. The comprehensive treatment methods have remained unchanged for more than 30 years. Surgery combined with chemotherapy can effectively treat 70% of osteosarcoma patients. However, chemotherapy resistance often occurs in recurrent cases, metastatic cases, and osteosarcoma patients who cannot be completely resected. Their 5-year DFS rate is less than 20% [5, 6]. However, a recent international study has shown that the survival rate of osteosarcoma does not improve with strengthened chemotherapy in high-risk groups [7].

Nomograms have been developed for most cancer types. A nomogram is more advantageous than the traditional staging system for numerous cancers [8–11]. Accordingly, it has been proposed as an alternative method and even as a new standard. To the best of our knowledge, the present study is the first attempt to establish a prognostic nomogram of osteosarcoma [11–13]. We used the data of osteosarcoma patient monitoring, epidemiology, and final outcome (SEER) database to analyse the impact of race, gender, and age on the OS of osteosarcoma patients, as well as to evaluate the degree of this impact. Furthermore, we established a prognostic nomogram to accurately predict individualised OS in patients with osteosarcoma and determine the appropriate treatment.

## 2. Methods

### 2.1. Patient Recruitment from the SEER Database

We retrieved the clinical variables of patients diagnosed with osteosarcoma from the SEER database (SEER × Stat 8.3.5). This database is a project set up by the National Cancer Institute to comprehensively perform national clinical investigation. The inclusion criteria were as follows: (1) bone (site recode, international classification of diseases for oncology (ICD-O-3)/WHO 2009); (2) complete TNM staging information; and (3) only one primary tumour selected. Finally, all cases included were randomly divided into training and verification sets. [14, 15].

### 2.2. Analysis of Clinical Variables

To establish the nomogram model, age, race, histological grade, sex, AJCC stage, AJCC TNM stage, and surgery were selected. With regard to clinical outcomes, OS was selected as the primary endpoint.

### 2.3. Construction and Validation of the Nomogram

Statistically, Cox was used for all classifications between the training and validation cohorts. Different variables were determined through univariate and multivariate analyses and further output. Then, a nomogram model was constructed with R software 4.0.2. The validation group was used to evaluate the newly established nomogram. The consistency index (C index) was used to evaluate the comparison between the nomogram prediction and observation results. A correction chart was used for visual comparison between nomogram prognosis-prediction map and real prognosis-prediction map. Sensitivity and specificity were evaluated using the receiver operating characteristic curve–area under curve. They were then compared with TNM and AJCC stages. All analyses were performed with R software 4.0.2, with *p* value < 0.05 as the difference to indicate statistical significance.

## 3. Results

### 3.1. Characterisation of Cases

Based on inclusion criteria, this study finally included a total of 2380 cases, in which 1669 cases were randomly assigned to the training cohort and 711 cases were assigned to the validation cohort (Table 1). Amongst the patients, 55.63% were female and 44.36% were male; 58.82% were less than 50 years old, 14.20% aged 50-59, 13.03% aged 60-69, 8.8% aged 70-79, and 52% aged over 80 years. Pathological stages I-IV accounted for 39.92%, 46.22%, 1.1%, and 12.77%, respectively. Grades 1-4 accounted for 18.15%, 23.28%, 22.94%, and 35.63%, respectively. Survival time of more than five years accounted for 7.1%, and survival time less than five years accounted for 92.94%. Stages T1-T3 accounted for 52.52%, 44.96%, and 2.5%, respectively. Stage M0 accounted for 88.24%, and stage M1 accounted for 11.76%. Stage N0 accounted for 97.86%, and stage N1 accounted for 2.16%. Surgery performed accounted for 88.78, and surgery not performed accounted for 11.22%.

### 3.2. OS of the Training Cohort

The OS median was 22 months. The 1-, 3-, and 5-year OS rates were 67.17%, 21.28%, and 6.65%, respectively.

### 3.3. Independent Prognostic Factors of the Training Cohort

Our study indicated that age, histological grade, pathological stage, and surgery performed were independent risk factors for the OS of osteosarcoma patients (Table 2) after performing multivariate analyses.

### 3.4. OS Prognosis Nomogram

**Figure 1:**
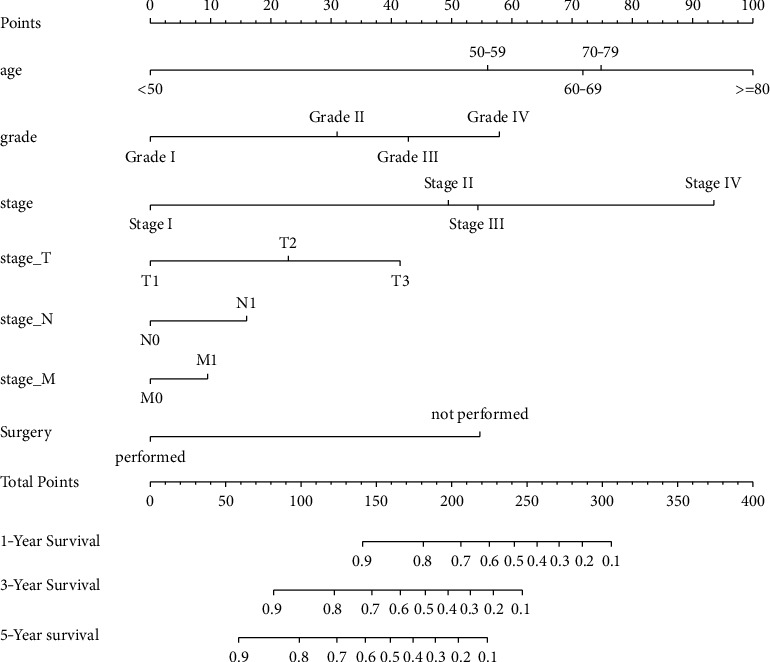
Osteosarcoma survival diagram. When using a nomogram, the value of each patient is on each variable axis, and a line is drawn up to determine the number of points received by each variable value. The sum of these numbers is on the total point axis, and a line is drawn down to the survival axis to determine the possibility of 1-, 3-, or 5-year survival.

**Figure 2:**
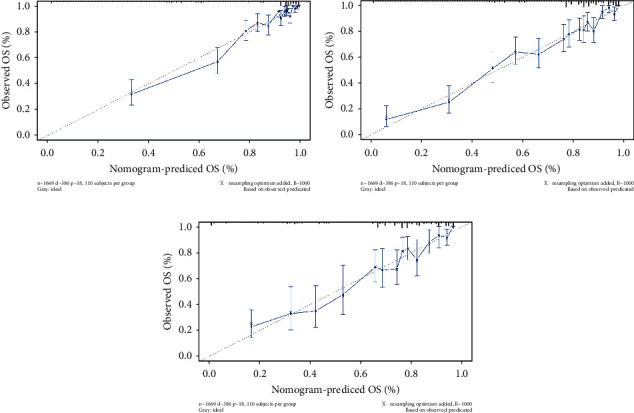
Calibration curves for training cohort to predict patient survival: (a–c) 1, 3, and 5 years. The nomogram (the predicted probability of overall survival (OS)) is plotted on the *X*-axis. The actual OS is plotted on the *Y*-axis.

**Figure 3:**
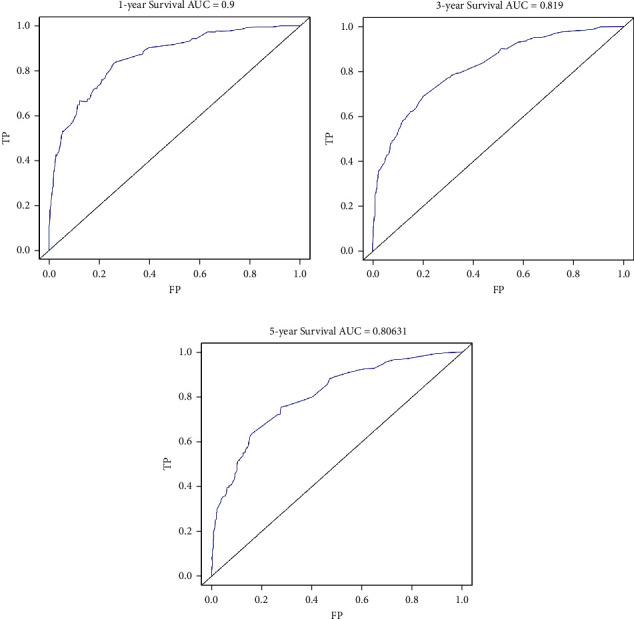
Comparison of receiving operating characteristic curve (ROC): (a) 1-year ROC of the training cohort, (b) 3-year ROC of the training cohort, and (c) 5-year ROC of the training cohort.

In the training cohort, the prognostic nomogram integrated all factors for OS (Figure 1). The C index of OS was predicted to be 0.82. The correction chart of 1-year, 3-year, or 5-year survival after operation showed consistency between nomogram prediction and actual observation (Figures 2(a)–2(c)).  Figure 3 shows the ROC curve for OS of 1-year, 3-year, and 5-year, and the area under curve (AUC) were 0.9, 0.819, and 0.80631, respectively, indicating that the model was accurate.

### 3.5. Comparison of Prediction Accuracy between Single Independent Factor and Nomogram

The survival ratio of AJCC stage and age was higher than the risk ratio of other factors. To compare the predictive ability of nomogram and AJCC staging and age on the prognosis of bone cancer. According to AJCC stage and age, the C indices of OS were 0.72 and 0.66, respectively, which were lower than that of nomogram (0.74), as shown in Table 2.

### 3.6. Kaplan–Meier Survival Analysis

**Figure 4:**
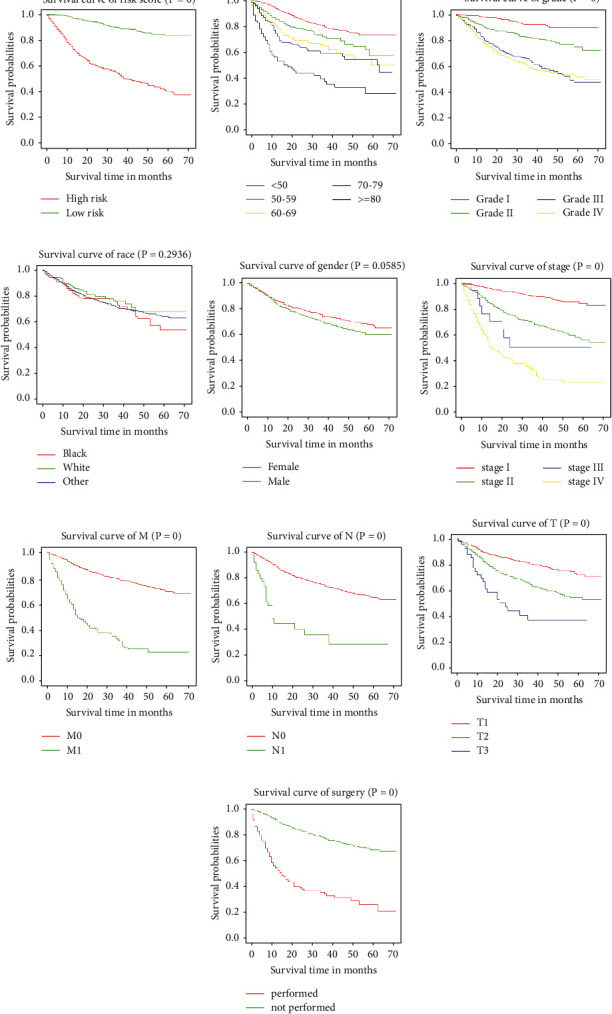
Kaplan–Meier survival analysis for patients with clinical features: (a) risk-score-dependent survival curve, (b) age-dependent survival curve, (c) grade-dependent survival curve, (d) race-dependent survival curve, (e) gender-dependent survival curve, (f) stage-dependent, (g) stage M-dependent survival curve, (h) stage N-dependent survival curve, (i) stage T-dependent survival curve, and (j) surgery-performed-dependent survival curve.

Kaplan–Meier survival analysis was conducted in a primary cohort to evaluate the impact of different statuses on the OS of osteocarcinoma patients. We then compared the relationship of clinical factors (age, sex, grade, race, T stage, M stage, N stage, clinical stage, and surgery performed) with patient prognosis. Results showed significant differences in survival rates between high- and low-risk groups in patients, M stage, N stage, T stage, grade, clinical stage, and surgery performed, as shown in Figure 4.

### 3.7. Validation of Predictive Accuracy of the Nomogram for OS

**Figure 5:**
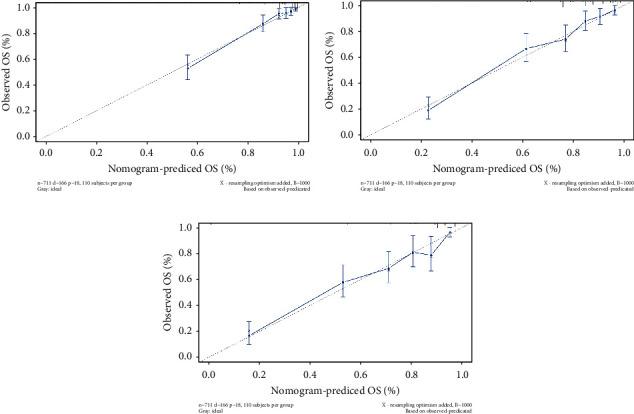
Calibration curves for validation cohort to predict patient survival: (a–c) 1, 3, and 5 years. The nomogram (the predicted probability of overall survival) is plotted on the *X*-axis. The actual overall survival is plotted on the *Y*-axis.

**Figure 6:**
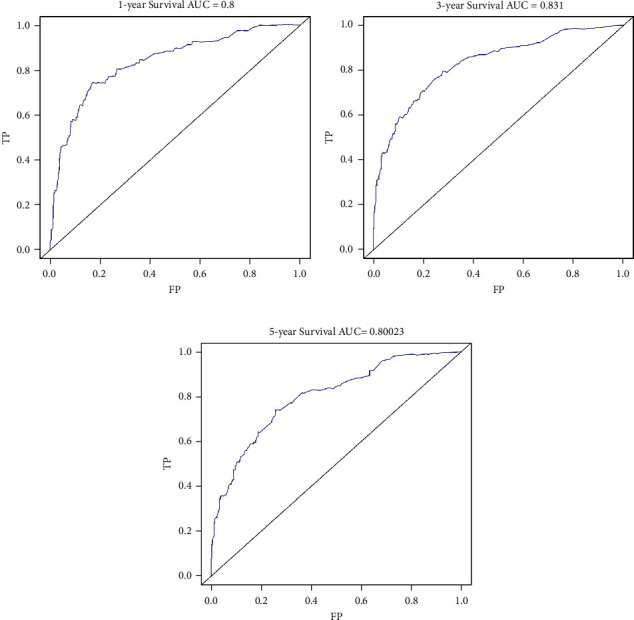
Comparison of receiving operating characteristic curve (ROC): (a) 1-year ROC of the validation cohort, (b) 3-year ROC of the validation cohort, and (c) 5-year ROC of the validation cohort.

In the validation cohort, the median OS time was 22 months (range = 0-71 months). The nomogram C index of predicted OS was 0.82, and the calibration curve displayed a good agreement between prediction and observation in the probability of 1-year, 3-year, and 5-year survival (Figure 5). The C-indices for OS prediction were 0.71 and 0.62 by AJCC stage and age, respectively, which were lower than that by the nomogram (0.82).  Figure 6 shows the ROC curve for OS of one year, three years, and five years, and the area under curve (AUC) were 0.8, 0.831, and 0.80023, respectively, indicating that the model was stable and reliable. After multivariate analysis, age, sex male, AJCC stage, stage T, and surgery were independent risk factors for OS in osteosarcoma patients (Table 3), which is basically consistent with the modeling results.

## 4. Discussion

With the emphasis on evidence-based medicine and precision medicine, increased attention is being paid to the value of data. Nowadays, the acquisition of large data and the transmission of information are developing more rapidly than before, and personalised medical treatment has become possible [16]. As a tool for assessing risks and benefits, clinical-prediction models can provide more intuitive and rational information to clinicians, patients, and administrators engaged in public-health undertakings. It has great potential in bone tumour prevention, treatment, and related research. Using CPMS can integrate multivariate information for unified analysis, and guiding clinicians to make treatment decisions is conducive to the prognosis of patients [17, 18]. However, a previous study has shown that although CPMS has a good ability to predict results, the frequency of clinicians using CPMS as a decision-making tool remains very low [5] [19]. In the clinical work of orthopaedic tumour diseases, doctors or patients generally make medical decisions based on relevant experiences or instructions of superior doctors. If objective and scientific reference is available, it would help orthopaedic doctors formulate medical plans and bring great benefits to patients.

CPMS has also been studied in orthopaedic-related tumours and metastases. With an incidence rate of 0.0004%-0.0005% in children and adolescents, osteosarcoma is a common primary malignant bone tumour [20, 21]. Osteosarcoma is characterised by high malignancy, rapid growth, difficult early diagnosis, and poor prognosis. The mortality and disability rates remain high, bringing a huge burden to patients, their families, and society [22, 23]. Early identification of high-risk potential osteosarcoma patients can help improve the prognosis of patients. A nomogram, which is an individualised prediction model for osteosarcoma prognosis, can be constructed. By collecting the relevant data of osteosarcoma patients for 10 years, the prediction model analyzes five independent prognostic factors. Compared with the traditional Enneking staging and AJCC staging system, it is more direct and accurate for realising the individualised prediction of osteosarcoma patients. In addition to improving the prediction accuracy, each patient can calculate their own survival rate, which provides important information for out-of-hospital prevention and follow-up monitoring.

Although multidisciplinary combined therapy significantly improves the survival rate of osteosarcoma, the existence of tumour metastasis causes osteosarcoma to be an incurable disease. Relevant studies have found that the survival rate of osteosarcoma with metastasis is about 20% [24]. Therefore, screening patients with high-risk osteosarcoma metastasis as soon as possible has a great clinical value. Some scholars have developed a nomogram to predict the metastasis probability of Enneking II B stage limb osteosarcoma after operation, which has high performance and versatility to predict the metastasis probability of Enneking II B stage limb osteosarcoma [25]. The development of nomograms is bound to contribute to the individualised assessment of the risk of osteosarcoma metastasis. With the introduction of combined systemic chemotherapy, the long-term survival rate of osteosarcoma patients has been significantly improved. Unfortunately, despite the numerous clinical trials over the past few decades, the survival rate of patients has not improved significantly. Osteosarcoma has obvious heterogeneous clinical behaviour. Up to 15% of patients can be cured by surgery alone [26]. Kim et al. [27] developed a nomogram to predict the neoadjuvant chemotherapy of AJCC stage II limb osteosarcoma and the prognosis of 5-year metastasis probability after definitive surgery. This figure can be used for personalised risk assessment and as the basis of risk adaptive treatment.

Nearly 90% of patients with osteosarcoma are reportedly classified as high-grade osteosarcoma at the time of diagnosis [28]. The 5-year OS is 45%-75%. Many treatments including surgery and adjuvant chemotherapy are beneficial to patients who may show poor survival, but not all patients with osteosarcoma can benefit from these treatments [29, 30]. Some clinical factors such as age, tumour volume, stage, histological subtype, and pathological fracture are correlated with the treatment results [31, 32]. Wu et al. [32] extracted the radiological features from the computed tomography images of preprocessed diagnosis, calculated the radiomics score of each patient by using the radiomics features to reflect survival probability, and established a nomogram to predict the 5-year expected survival rate of osteosarcoma patients in combination with the radiomics score and clinical factors. A nomogram combines the radiological characteristics and clinical factors, improves the prediction accuracy compared with the previous prediction model, and becomes an independent part. A nomogram is also an early survival prediction of osteosarcoma based on radiology. It can enable doctors to provide patients with more appropriate treatment strategies and arrange reasonable follow-up intervals to avoid unnecessary costs and consumption of medical resources.

In recent years, research on prediction models has played a positive role in helping orthopaedic doctors and patients select treatment methods, which can effectively lead to reduced medical costs. Owing to the patient's educational background and many other factors, patients often cannot understand the reasons for the choice of treatment plan. A nomogram's visual map has a great clinical value in such situations.

Clinical models should accurately predict specific events and be relatively simple and easy to use. If the prediction model provides inaccurate estimates of the occurrence of future events, it would mislead medical staff to make difficult treatment choices and insufficiently manage medical resources. Meanwhile, if a model has high predictability but is difficult to apply (e.g., calculating complex or unfamiliar problems, projects, or units), time consuming, expensive, or has low relevance (e.g., some of the above models were based on U.S. database resumes), it would not be widely used. Therefore, striking a balance between predictability and simplicity is the key to a good CPMS. A CPMS needs to be constantly updated and verified, but few external verification methods for predicted performance is available. Considering that model development is complex, consulting statistical experts can improve the effectiveness and quality of accurate prediction-model research. After a model is developed and before the model is applied to practice, multiple external data sets should be used for strong verification and effective dissemination to interested parties [18].

Conclusion, in the current work, we identified age, clinical stage, grade, surgery performed, and T stage as independent risk factors affecting the prognosis of patients with osteosarcoma by using a clinical prediction model. In our study, the training cohort and validation cohort were divided into seven or three points, which is more appropriate, because that if the proportion of the test set is relatively large, it may lead to a large difference between the evaluation model and the previous one; thus, reducing the fidelity of the evaluation. And in this study, we mainly focus on the impact of surgery on the prognosis of patients. Radiotherapy, chemotherapy, and resection methods are not the focus of this study, so surgical code, chemotherapeutic state, and tumor sequence code are not included. Nevertheless, this study has the following limitations: [1] no multicentre research report was used, and multisource data should have been externally verified before clinical application; and [2] most data originated from the SEER database. The database is supported by the National Cancer Institute, and 17 population-based cancer registries collect data on incidence rate and survival rate, accounting for 26% of the US population. However, the database does not collect detailed information on cancer treatment modalities and recurrence. The ethnic composition greatly differs from that of China and cannot be used there directly. To develop multicentre external validation and update the model promptly, the cooperation of multiple regions and hospitals is required to fully exploit the value of the prediction model.

## Figures and Tables

**Table 1:** Baseline demographic and clinical characteristics of patients with osteocarcinoma (continued).

Characteristic	Training cohort (*n* = 1669)		Validation cohort (*n* = 711)	
	No.	%	No.	%
Age				
<50	967	57.94	433	60.90
50–60	255	15.28	83	11.67
60–70	203	12.16	107	15.05
70–80	153	9.17	56	7.88
>80	91	5.45	32	4.50
Gender				
Female	921	55.18	403	56.68
Male	748	44.82	308	43.32
Pathologic stage				
I	690	41.34	260	36.57
II	750	44.94	350	49.23
III	19	1.14	7	0.98
IV	210	12.58	94	13.22
Grade				
G1	320	19.17	112	15.75
G2	398	23.85	156	21.94
G3	367	21.99	179	25.18
G4	584	34.99	264	37.13
Survival time				
Long (>5 years)	111	6.65	57	8.02
Short (<5 years)	1558	93.35	654	91.98
Stage T				
T1	876	52.49	374	52.60
T2	749	44.88	321	45.15
T3	44	2.64	16	2.25
Stage M				
M0	1477	88.50	623	87.62
M1	192	11.50	88	12.38
Stage N				
N0	1632	97.78	697	98.03
N1	37	2.22	14	1.97
Race				
Black	174	10.43	84	11.81
Other	134	8.03	63	8.86
White	1361	81.55	564	79.32
OS status				
Dead	386	23.13	166	23.35
Alive	1283	76.87	545	76.65
Surgery				
Performed	1481	88.74	632	88.89
Not performed	188	11.26	79	11.11

**Table 2:** Multivariate analysis of overall survival in the training cohort.

Variable	Exp (coef)	Exp (-coef)	Lower (95% CI)	Upper (95% CI)	*P*
Age 50–59	2.8415	0.3519	2.1012	3.8427	^*∗∗∗*^
Age 60–69	3.8223	0.2616	2.7993	5.2193	^*∗∗∗*^
Age 70–79	4.0643	0.246	2.9508	5.5979	^*∗∗∗*^
Age ≥80	6.5395	0.1529	4.5627	9.3726	^*∗∗∗*^
Sex male	1.1093	0.9015	0.8996	1.3678	
Race other	0.9185	1.0888	0.562	1.501	
Race white	0.9255	1.0805	0.671	1.2766	
Grade II	1.7774	0.5626	1.0418	3.0324	^*∗*^
Grade III	2.2618	0.4421	1.1054	4.628	^*∗*^
Grade IV	2.9943	0.334	1.4693	6.1019	^*∗∗*^
Stage II	2.4439	0.4092	1.3572	4.4007	^*∗∗*^
Stage III	2.658	0.3762	0.9055	7.8019	
Stage IV	5.5274	0.1809	1.9804	15.4276	^*∗∗*^
Stage T2	1.5134	0.6608	1.2113	1.8908	^*∗∗∗*^
Stage T3	2.1475	0.4657	1.2178	3.7868	^*∗∗*^
Stage N1	1.3666	0.7318	0.7322	2.5505	
Stage M1	1.1882	0.8416	0.4837	2.919	
Surgery performed	0.3576	2.7963	0.2779	0.4602	^*∗∗∗*^

**Table 3:** Multivariate analysis of overall survival in the validation cohort.

Variable	Exp (coef)	Exp (-coef)	Lower (95% CI)	Upper (95% CI)	*P*
Age 50–59	2.7954	0.3577	1.6499	4.7362	^*∗∗∗*^
Age 60–69	3.1459	0.3179	2.0462	4.8368	^*∗∗∗*^
Age 70–79	5.0593	0.1977	3.0214	8.4717	^*∗∗∗*^
Age ≥80	3.5395	0.2825	1.836	6.8233	^*∗∗∗*^
Sex male	2.0548	0.4867	1.4482	2.9155	^*∗∗∗*^
Race other	0.5716	1.7494	0.268	1.2195	
Race white	0.6514	1.5352	0.4135	1.0262	
Grade II	1.7897	0.5587	0.7909	4.0497	
Grade III	1.1684	0.8559	0.3277	4.1663	
Grade IV	1.1424	0.8754	0.3246	4.0202	
Stage II	4.534	0.2206	1.3659	15.0495	^*∗*^
Stage III	9.4941	0.1053	1.524	59.146	^*∗*^
Stage IV	9.1067	0.1098	1.6086	51.5548	^*∗*^
Stage T2	1.646	0.6075	1.1635	2.3286	^*∗∗*^
Stage T3	1.3239	0.7553	0.5496	3.1893	
Stage N1	0.7803	1.2816	0.3244	1.877	
Stage M1	1.6933	0.5906	0.3562	8.049	
Surgery performed	0.288	3.4718	0.195	0.4255	^*∗∗∗*^

## Data Availability

The original data could be obtained from the corresponding author upon request.
